# Rapid Optimization of External Quantum Efficiency of Thin Film Solar Cells Using Surrogate Modeling of Absorptivity

**DOI:** 10.1038/s41598-018-26469-3

**Published:** 2018-05-25

**Authors:** Mine Kaya, Shima Hajimirza

**Affiliations:** Department of Mechanical Engineering, Texas A&M University, 3123 TAMU, College Station, TX 77843-3123 USA

## Abstract

This paper uses surrogate modeling for very fast design of thin film solar cells with improved solar-to-electricity conversion efficiency. We demonstrate that the wavelength-specific optical absorptivity of a thin film multi-layered amorphous-silicon-based solar cell can be modeled accurately with Neural Networks and can be efficiently approximated as a function of cell geometry and wavelength. Consequently, the external quantum efficiency can be computed by averaging surrogate absorption and carrier recombination contributions over the entire irradiance spectrum in an efficient way. Using this framework, we optimize a multi-layer structure consisting of ITO front coating, metallic back-reflector and oxide layers for achieving maximum efficiency. Our required computation time for an entire model fitting and optimization is 5 to 20 times less than the best previous optimization results based on direct Finite Difference Time Domain (FDTD) simulations, therefore proving the value of surrogate modeling. The resulting optimization solution suggests at least 50% improvement in the external quantum efficiency compared to bare silicon, and 25% improvement compared to a random design.

## Introduction

The solution of inverse problem is challenging when analytical solution is not available for the forward problem. This is the case for many engineering problems with underlying physical utilities and constraints where the *best design* is sought. Underlying many complex design problems reside spatially and temporally discretized partial differential equations, which usually do not render closed form solutions. Consequently, there is no explicit expression for the design utility function (i.e. forward problem) and its derivatives (i.e., gradient vector, Hessian matrix, etc.). Therefore, inverse problem can only be solved within a black-box optimization framework which requires many iterative simulations of the forward problem and thus can be computationally expensive^1–3^. The progress in computational methods and resources has made solving complex design problems feasible by means of more powerful simulators, parallel processing and more to-the-point black box optimization algorithms.

An example of a computationally expensive problem with non-explicit derivative is modeling opto-electrical characteristics of devices at subwavelength scales, including thin film solar cells (TFSC). Governing equations for this problem are Maxwell’s electromagnetic equations which are partial differential equations expressing the relationship between electric and magnetic fields and current flow, as well as drift-diffusion equations that determine the transport of carriers inside a medium. In order to model the efficiency of a solar cell in response to solar irradiance, Maxwell’s equations must be solved to render the number and distribution of absorbed photons in the absorber layer (semiconductor). Since solving Maxwell’s equations for a relatively wide solar spectrum (visible, UV and IR) is time-consuming given the current numerical technologies, TFSC design problem is limited to heuristic searches, intuitive guesses and experimental evaluations. Recent research work, including the efforts of the author, has attempted to utilize smart and efficient global optimization algorithms to systematically design efficient TFSCs^4–11^.

An alternative to costly black-box optimization is surrogate modeling. The unknown black-box function can be approximated using a learning model by collecting a representative set of training data. The resulting model is presumably much more efficient to calculate for a new set of input parameters than the original forward problem^12^. The surrogate model does not have to be the complete black-box function, but rather a subcomponent function. Specifically, if the utility function is known to be a sum of another sub-function over a range of a few input parameters, then a more efficient approach is to approximate the sub-function. This happens to be the case for TFSCs; the number of absorbed photons is the weighted integral of optical generation rate (absorptivity) at individual wavelengths, therefore single frequency optical characteristics can be modeled and approximated^13–15^.

In the present work, we use surrogate modeling for approximating optical absorptivity, and therefore external quantum efficiency of a particular class of multi-layered thin film solar cells. We use the learned models to efficiently design the multi-parameter geometry of the TFSC structure for maximum efficiency. The multi-layered silicon-based TFSCs that we study consist of front anti-reflective and metallic back-reflector coatings, and have practical thin oxide layers that occur during the fabrication process. We demonstrate that neural networks (NN) can be used to reliably learn the optical absorptivity of the structures as a function of geometry and radiation wavelength. Additionally, we show that the resulting optimization reduces the computational burden of optimization by a factor of 5 to 20. The resulting design shows 25% improvement in the external quantum efficiency compared to a random design, and 50% improvement compared to bare silicon.

This paper is organized as follows. First an overview of the multilayer solar cell structure and fundamentals of optical modeling in subwavelength scale solar cells are presented. Then the framework of the present optimization problem along with the basics of surrogate-based optimization and regression models are provided. Finally, numerical results are presented and discussed.

## Thin Film Multilayer Solar Cell Structure

**Figure 1:**
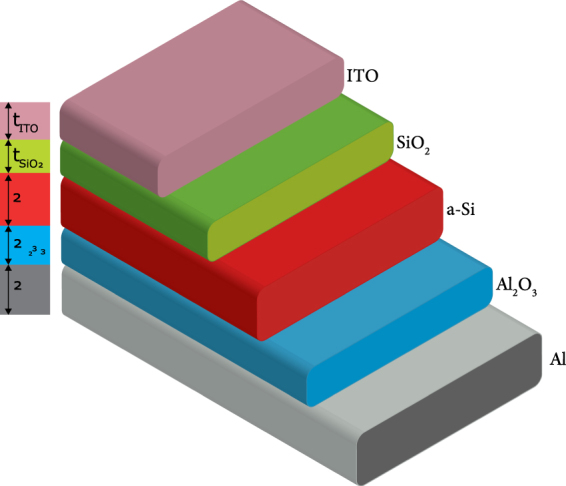
Thin film multilayer solar cell structure.

The thin film solar cell structure in Fig. 1 is considered in the present study. The amorphous silicon (a-Si) active layer is stacked between aluminum back reflector and antireflective indium tin oxide (ITO) layers. The oxides Al_2_O_3_ and SiO_2_ formed during manufacturing could help to improve solar cell performance by balancing the refractive indices between a-Si and the front/back layers^7,16^.

The performance of a solar cell is often quantified by internal (IQE) and external (EQE) quantum efficiencies. IQE is the ratio of electrons collected as photocurrent to the number of absorbed photons. On the other hand, EQE combines optical performance and charge generation and calculates the ratio of the number of electrons collected as photocurrent to the number of photons incident to the solar cell. Ideal EQE is unity, however this is never the case due to two main practical reasons: (i) optical losses due to photons that are not absorbed and (ii) recombination losses due to non-collected excitons^17^. EQE is calculated from Eq. (1).

1 $${{\eta }}_{{e}}=\frac{{{N}}_{{e}}}{{{N}}_{{i}}}=\frac{{{N}}_{{p}}}{{{N}}_{{i}}}\frac{{{N}}_{{e}}}{{{N}}_{{p}}}={{\eta }}_{{A}}\,{{\eta }}_{{I}},$$

where *η*_*A*_ = *N*_*p*_/*N*_*i*_ is the absorption efficiency, *N*_*i*_ is the number of photons incident on the solar cell and *N*_*p*_ is the photons absorbed within a-Si layer given by:

2 $${N}_{p}=\frac{1}{hc}\int \lambda \,\alpha (\lambda )\,I(\lambda )d\lambda ,$$

3 $${N}_{i}=\frac{1}{hc}\int \lambda \,I(\lambda )d\lambda .$$

where *h* is the Planck’s constant, *c* is the speed of light, *α*(*λ*) is the spectral absorptivity and *I*(*λ*) is the irradiance spectrum. In this study, AM1.5 standard terrestrial spectrum^18^ is used for irradiance.

*N*_*e*_ is the number of electrons collected as photocurrent, and *η*_*I*_ is IQE. *η*_*I*_ is calculated from:

4 $${\eta }_{I}=\frac{{N}_{e}}{{N}_{p}}=\frac{{J}_{L}/q}{{N}_{p}}=\frac{1}{{N}_{p}}\frac{1}{q}[q{\int }_{0}^{{t}_{aSi}}{n}_{p}(x){{\mathbb{P}}}_{c}(x)dx].$$

where *J*_*L*_ is the photocurrent, *q* is the elementary charge, *n*_*p*_(*x*) is the carrier generation rate, ${{\mathbb{P}}}_{c}$ is *collection probability* defined as the probability that a carrier generated due to absorption contributes to the photocurrent.

*n*_*p*_(**x**) depends on the number of photons absorbed within the active layer. Assuming photons are absorbed uniformly within a-Si layer, *η*_*I*_ becomes:

5 $${\eta }_{I}=\frac{1}{{t}_{aSi}}{\int }_{0}^{{t}_{aSi}}{{\mathbb{P}}}_{c}(x)dx.$$

**Figure 2:**
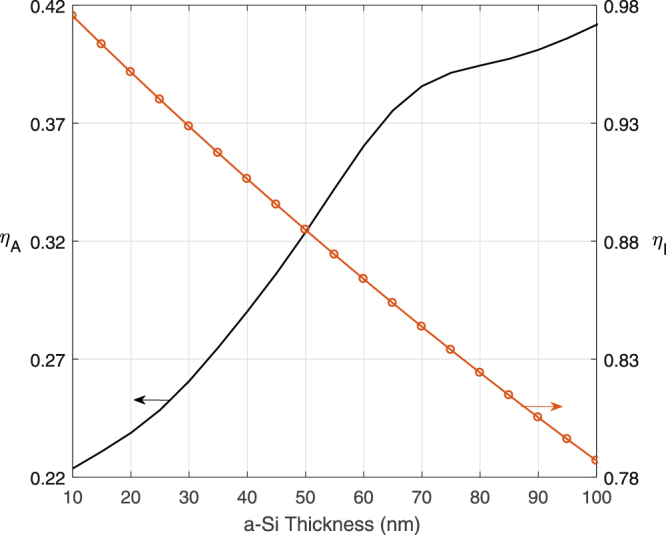
The variation of *η*_*A*_ and *η*_*I*_ with respect to *t*_*aSi*_.

IQE and EQE are mostly experimentally measured in order to calculate short-circuit current density^19^. There are numerous examples of experimental measurements of IQE and EQE for solar cells. For example, Thouti *et al*.^20^ measured IQE of a textured silicon solar cell to estimate the effective diffusion length due to the presence of resonance effect of silver nanoparticles. There have also been attempts at obtaining analytical expressions for the quantity. Ferrero *et al*.^21^ proposed a method to calculate EQE of photodiodes based on Beer’s law for absorption and Shockley-Read-Hall recombination. The structure is divided into front, space-charge (depletion) and rear regions and treated differently regarding recombination. Dibbs *et al*.^22^ approximated the collection model as a step function, which is unity in the depletion region and zero elsewhere. In these two theoretical models, the exact knowledge of the dimensions of depletion zone and p and n layers are required. A probabilistic model of EQE similar in principle to those of Xue *et al*.^23,24^ is used in this work which preserves the dependence of collection probability to absorber thickness and diffusion length. The essence of the model is as follows: In general, in a p-n junction solar cell, ${{\mathbb{P}}}_{c}$ is unity in the depletion region and decreases exponentially as the distance from depletion region increases^21,22^. Assuming surface recombination is negligible compared to bulk recombination, ${{\mathbb{P}}}_{c}$ can be approximated as:

6 $${{\mathbb{P}}}_{c}(x)=\exp (-\frac{|x-{t}_{aSi}/2|}{{L}_{D}}),$$

where *L*_*D*_ is the diffusion length of the carriers. After substituting (2–6) in Eq. (1), *EQE* becomes,

7 $${\eta }_{e}=\frac{{N}_{p}}{{N}_{i}}\frac{1}{{\tilde{t}}_{aSi}}(1-{e}^{-{\tilde{t}}_{aSi}}),$$

where $\tilde{t}$_*aSi*_ = 0.5*t*_*aSi*_/*L*_*D*_ . *L*_*D*_ is ~100 nm for amorphous silicon^25^. Note that according to (1), EQE has two components: an optical component (*η*_*A*_) which has a complicated tie to all geometry parameters and material choices, and can only be determined via solving the Maxwell’s equations. The second component (*η*_*I*_) is IQE and is only a function of silicon height and junction diffuse length in this simplified model. Generally, the expectation is that thicker silicon results in higher absorption, but also higher recombination, thus lower electric factor. The trade-off between these two components is the key to optimal design. This trade-off is shown for a random fixation of all geometry parameters but varying silicon thickness in Fig. 2.

**Figure 3:**
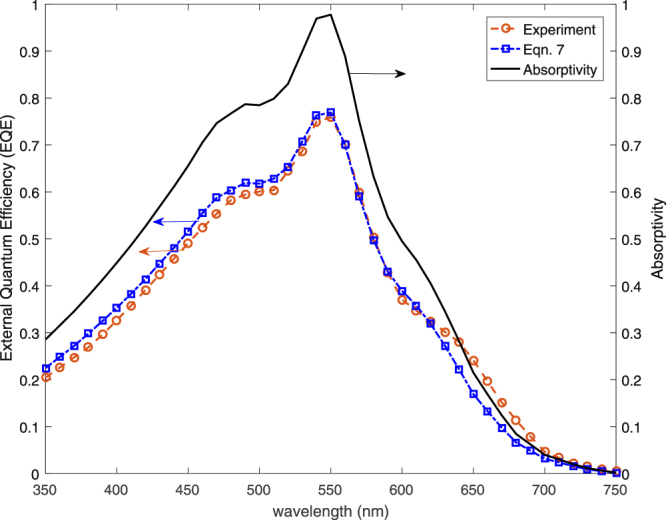
Comparison of measured and calculated *EQE* and simulated absorptivity profile for Ag/ZnO:Al/a-Si/ITO solar cell.

The proposed EQE model is validated with experimental results in the literature^26^. The authors measured EQE of a Ag/ZnO:Al/a-Si/ITO solar cell and calculated absorbed power in the a-Si layer using FDTD method. The same absorption profile is used to calculate EQE using Eq. (7) for *t*_*aSi*_ = 100 *nm* and *L*_*D*_ = 100 *nm*. The comparison of experiments and present calculations based on absorptivity is given in Fig. 3. Note that the model matches closely with the experiments for most of the relevant spectrum.

## Optimization

The aim of the present study is to maximize EQE given in Eq. (7) by modifying the solar cell geometry vector, **x**:

8 $$\begin{array}{c}{{\rm{\max }}}_{{\bf{x}}}{\eta }_{e}({\bf{x}}),\\ {{\bf{x}}}_{{\bf{L}}} < {\bf{x}} < {{\bf{x}}}_{{\bf{U}}},\end{array}$$

where **x**_***L***_ and **x**_***U***_ are lower and upper bounds imposed on **x**, respectively. The present optimization problem requires computation of *N*_*p*_ at every iteration (Eq. (2)), where the integrand is evaluated at a full spectrum of irradiance wavelengths, and then numerically integrated. When the step size for the wavelength is 10 nm, *α*(*λ*) must be calculated 46 times (within the 300–750 nm wavelength range) using FDTD to complete one iteration of the optimization. A surrogate for *α*(*λ*) could accelerate the optimization procedure provided that it predicts the output accurately.

### Surrogate-Based Optimization

Surrogate-based optimization (SBO) provides a faster way to maximize a black-box utility function that requires expensive computations. Examples of time-consuming objective functions based on underlying first principle calculations include fluid dynamics equations, heat transfer equations and Maxwell’s electromagnetic equations as in the present study. In SBO, a regression model is fit to the objective function as an approximate model, using past data. The key to the merit of SBO is the accuracy and reliability of the regression (surrogate) model. Once the surrogate model is trained with the data pool obtained by performing a limited number of computationally expensive simulations, and after the accuracy and reliability of the model is verified, the optimization task could be accomplished efficiently.

For the present problem of maximizing EQE, referring to Eqs (1–7) we can express EQE for an arbitrary geometry **x** as:

9 $${\eta }_{e}({\bf{x}})=\frac{a(1-{e}^{-b{x}_{3}})}{{x}_{3}}\int \lambda \,f({\bf{x}},\lambda )\,I(\lambda )d\lambda ,$$

where $a=\frac{2{L}_{D}}{hc{N}_{i}},\,b=\frac{0.5}{{L}_{D}}$ are constants, *x*_3_ is the third coordinate of **x**, namely *t*_*aSi*_, and *f*(**x**, *λ*) is the spectral absorptivity for the geometry **x** at wavelength *λ*. The most computationally efficient way to approximate *η*_*e*_ is therefore to approximate spectral absorptivity with a surrogate function:

10 $$\hat{f}({\bf{z}})\approx f({\bf{z}}).$$

Therefore the surrogate-based optimization becomes:

11 $$\begin{array}{c}{\max }_{{\bf{x}}}{\hat{\eta }}_{e}({\bf{x}}),\\ {{\bf{x}}}_{{\bf{L}}} < {\bf{x}} < {{\bf{x}}}_{{\bf{U}}},\end{array}$$

where

12 $${\hat{\eta }}_{e}({\bf{x}})=\frac{a(1-{e}^{-b{x}_{3}})}{{x}_{3}}\int \lambda \,\hat{f}({\bf{x}},\lambda )\,I(\lambda )d\lambda ,$$

The state-of-the-art surrogate modeling techniques are rooted in the machine learning literature and include Polynomial Regression (PR), Support Vector Machines (SVM), Gaussian Process (GP) (also known as Kriging) and Neural Networks (NN). Depending on the nature of the problem, at least one of these techniques could successfully be used to map the input space to the output space. NN is a well-established regression tool and is noteworthy for its ability to approximate almost every function regardless of the degree of nonlinearity^27,28^. When engaged with regularization techniques, it is possible to obtain reliable models without overfitting. Basics of modeling using NNs are provided below and more in Supplementary Information.

#### Neural Networks

Neural Networks (NN) are machine learning tools that can approximate input-output relationships of complex functions by mimicking the neuro-chemical brain learning process. A NN consists of a series of multiple layers of functional relationships evaluated at parallel perceptron units (neurons). More details on the mathematical formulation of NN, training and regularization for the specific utility function of this work are presented in Supplementary Information.

#### Design of Experiments and Validation

Regardless of what regression method is used, there are two important factors which determines the success of surrogate modeling: design of experiments and validation^29^. Design of experiment means sampling to determine where on input space the actual simulations will be performed. There are several available sampling options, such as Latin hypercube sampling (LHS), orthogonal arrays (OA)^29^ and uniform sampling (US). In this study, the input space is sampled uniformly. Validation is the other important step of SBO, which provides an instantaneous estimate of the out-sample performance of the model during training. Some portion of the available data (validation set) is not introduced to the training, but rather used only for evaluating the model out-sample performance. The validation set can be obtained from the data pool in several ways. In the split-sample (SS) method, training and validation sets are divided only once, and training stops if validation error starts increasing. On the other hand, cross validation (CV) or leave-one out (LOO) method splits the data *n* times (*n*:number of folds) and every one of *n* folds are used as validation set while the rest is used for model fitting. Although CV requires *n* times training, it usually renders an unbiased model. CV is used in the current work for model learning.

### Algorithms

Optimization of the objective function could be done by means of deterministic and heuristic methods. Deterministic methods, such as Steepest Descent (SD), Conjugate Gradient (CG) and Quasi-Newton (QN), rely on the gradient and Hessian of the objective function. Although these methods have guaranteed convergence for convex functions, they have the tendency of local entrapment for highly nonconvex functions and thus often only yield a local optimum. Therefore, they are usually used in conjunction with another global heuristic method. The appealing factor about QN is that when NNs are used as surrogate functions, an explicit expression for the gradient of the surrogate utility function is available, thus the QN can be implemented very efficiently.

Heuristic methods utilize random search which are unlikely to trap at local optima. These methods include Tabu Search (TS), evolutionary algorithms, population based algorithms and Simulated Annealing (SA). In this study, we use a combination of Simulated Annealing and Quasi-Newton (QN) algorithms for NN surrogate function. First a SA global optimizer is used to achieve a near optimal solution. Then QN is used to locally improve the search. Details of these algorithms are explained in Supplementary Information.

## Results and Discussion

### Data Generation

The aim of the surrogate model is to establish a functional relationship between the absorbed power in the active layer, solar cell geometry and wavelength of the incoming light. As discussed in the previous section, a pool of data is required for training the surrogate. Data pool is generated by sampling from the input space, then running FDTD simulations on the data and obtaining output values (see Section 6 on Methods). Lower and upper bounds are imposed to the input vector in order to restrict our focus to a reasonable limited range. These bounds are shown in Table 1.

**Table 1:** Lower (LB) and upper (UB) bounds for the multilayer solar cell.

Parameters	*t*_*ITO*_	${{\boldsymbol{t}}}_{{\boldsymbol{Si}}{{\boldsymbol{O}}}_{{\bf{2}}}}$	*t*_*aSi*_	${t}_{{\boldsymbol{A}}{{\boldsymbol{l}}}_{{\bf{2}}}{{\boldsymbol{O}}}_{{\bf{3}}}}$	*t*_*Al*_	*λ*
LB (nm)	20	5	20	5	20	300
UB (nm)	80	20	200	20	120	750

A total of 1000 points are uniformly sampled from the input space, and the corresponding output values (absorptivity) are obtained from FDTD simulations. These 1000 simulations are equivalent to ~22 direct simulations for absorptivity of distinct geometries when simulated over the wavelength range with 10 nm increments.

### Model Fitting

**Figure 4:**
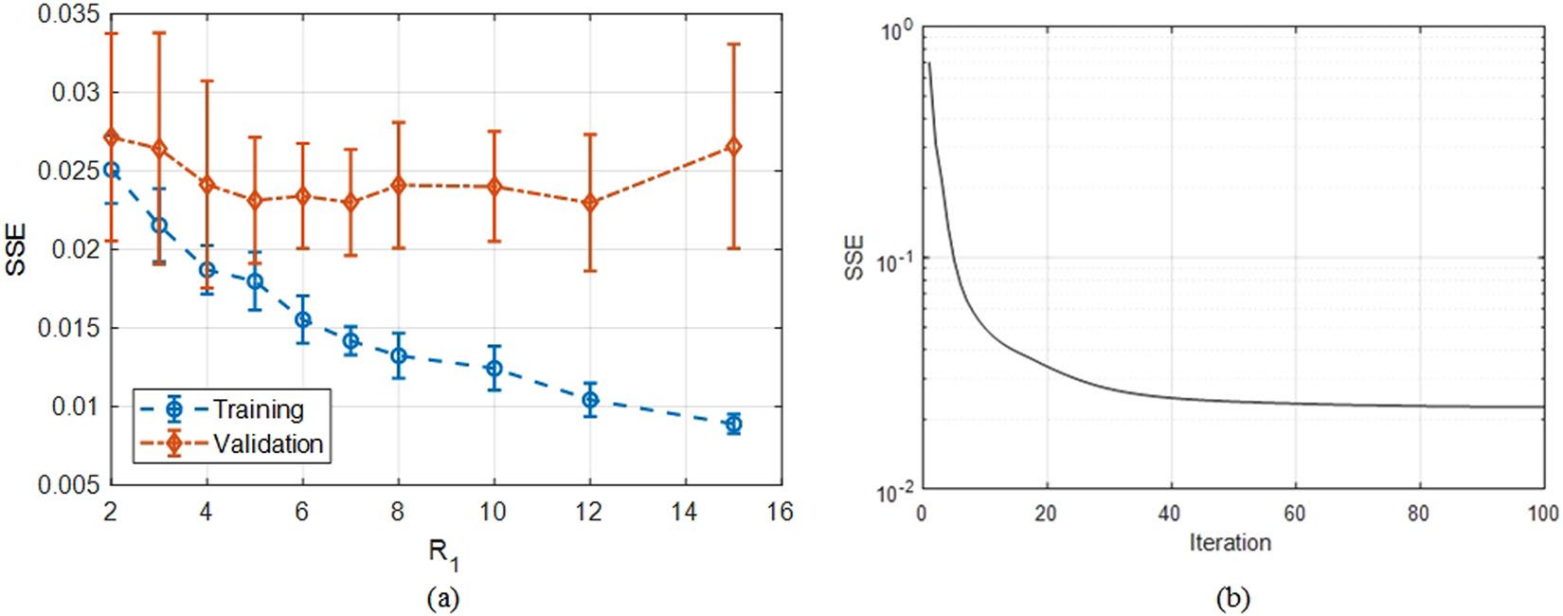
Neural network training. (**a**) Normalized mean sum of squared error (SSE) with respect to number of neurons in hidden layer (*R*_1_), (**b**) Evolution of SSE cost function during final NN training.

NN architecture is 6-*R*_1_-1 where *R*_1_ is the number of neurons in the hidden layer and is determined based on minimum validation error principle: Cross validation is used during training, and mean training and validation errors are calculated as the average of *n* = 4 folds. Optimal *R*_1_ is where the validation error does not improve despite increasing *R*_1_. The results for the NN training are presented in Fig. 4. The optimal *R*_1_ is determined as 7 in this case (see Fig. 4a). The final coefficient matrices are then obtained by training the NN with this configuration over the entire available data. The variation of Sum Square Error (SSE) during the final training is presented in Fig. 4b. The trained model is then used as the surrogate for FDTD simulations in optimization, the results of which will be demonstrated in the next section.

### Optimization

The trained NN surrogate model is used instead of original FDTD simulations for designing the optimal structure. The search is done using two randomly selected starting points of x_*i*,1_ = [50, 12, 110, 12, 70]^*T*^ and x_i,2_ = [20, 10, 70, 10, 60]^*T*^. Every search includes a SA optimization followed by a local QN optimization starting at the optimal point of SA. Final optimization results are presented in Table 2, and are compared with the optimization of the previous study^7^. The reference^7^ used a regression-tree based optimizer as well as SA on direct FDTD simulations to find the optimal solution. However, since the objective function in this study^7^ is slightly different than the present objective function, a deviation between the results of these two studies is expected. The present study achieved a slightly higher EQE than that in the reference^7^.

**Table 2:** Results of optimization.

Solution	*t*_*ITO*_	${{\boldsymbol{t}}}_{{\boldsymbol{Si}}{{\boldsymbol{O}}}_{{\bf{2}}}}$	*t*_*aSi*_	${{\boldsymbol{t}}}_{{\boldsymbol{A}}{{\boldsymbol{l}}}_{{\bf{2}}}{{\boldsymbol{O}}}_{{\bf{3}}}}$	*t*_***Al***_	*η*_*e*_ (NN)	*η*_*e*_ (FDTD)
1. NN-SA-QN	29	19	65	20	101	0.356	0.370
2. NN-SA-QN	29	20	65	20	98	0.356	0.369
Reference^7^	30	16	62	20	50	0.350	0.361

**Figure 5:**
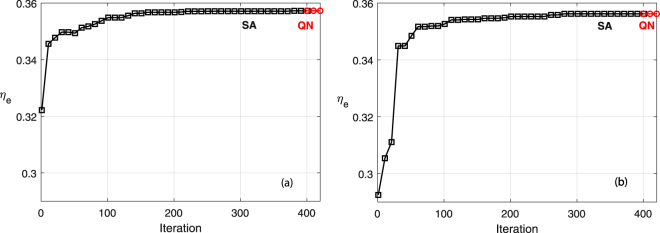
Evolution of surrogate EQE during optimizations with initial guesses (**a**) **x**_1_, (**b**) **x**_2_.

The required time to complete optimization for the corresponding algorithms are also recorded. Each SA algorithm is iterated 400 times, and the consequent QN takes several extra iterations. NN-based SA-QN takes 30 seconds to find an optimal solution, which is negligible compared to the amount of time spent at finding the true EQE value for a given geometry using FDTD (~600 seconds). The overall number of computations is equivalent to only 20 full-spectrum FDTD calculations, which is impressively smaller than approximately 100 iterations in regression-tree-based search, and 200–400 iterations in SA on direct FDTD calculations used in the reference study^7^. Therefore, the overall computation time is reduced by a factor of 5–20 using the proposed surrogate-based optimization. The evolution of EQE during the iterations for the results presented in Table 2 is shown in Fig. 5.

**Figure 6:**
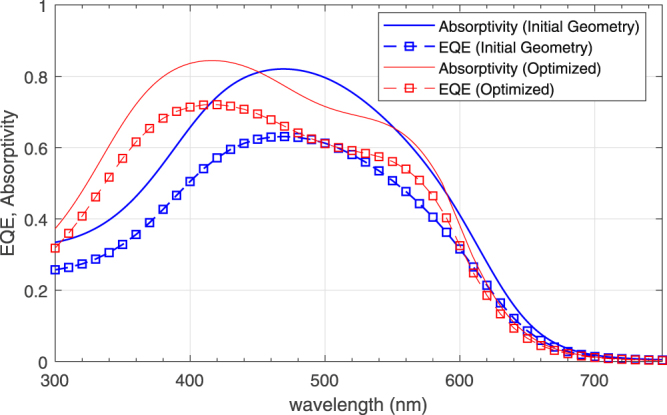
Absorptivity and EQE of initial (**x**_1_) and optimized geometries in Table 2.

The absorptivity and EQE spectrum of the initial solution **x**_1_ and the one obtained by optimization starting from **x**_1_ are shown in Fig. 6. Note that the majority of improvement is due to a wider absorption spectrum in the case of the optimal solution, especially at wavelengths shorter than 450 nm. The optimal solution has more than 25% improvement in efficiency, is more broadband and has larger optical absorption despite a thinner silicon layer. The optimal solution has also 50% more EQE compared to optimal thickness bare silicon (~50 nm^7^).

**Figure 7:**
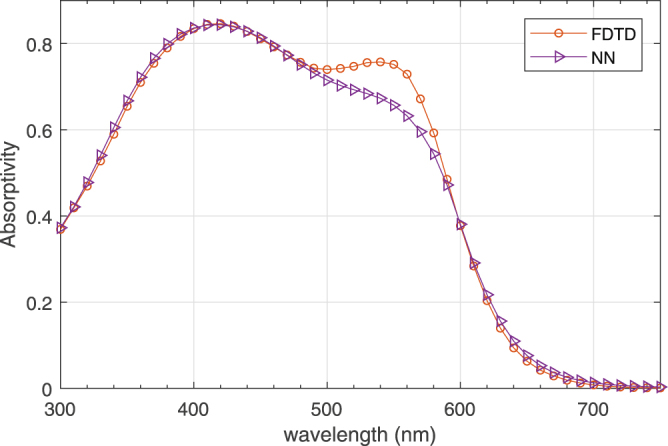
Absorptivity of optimized geometry obtained by NN and FDTD.

The (surrogate) spectral absorptivity of optimized geometry is also compared with the results from FDTD for the same geometry in Fig. 7. Note that the profiles are in a good agreement in most of the spectrum except *λ* = 500−600 nm which is the reason of the discrepancy between *EQE* values obtained by NN and FDTD.

## Conclusion

We demonstrated that the wavelength-specific optical absorptivity of a thin film multi-layered amorphous-silicon-based solar cell can be modelled accurately with NNs, and can be efficiently approximated as a function of cell geometry and wavelength. Using this framework and modeling the external quantum efficiency of the cell as a function of absorptivity and electrical recombination factor, we optimized a multi-layer thin film solar cell structure consisting of ITO front coating and metallic back-reflectors and oxide layers for maximum efficiency. Our required computation time for an entire model fitting and optimization was 5 to 20 times faster than the optimization times of the best previous results, therefore proving the value of surrogate modeling. The final design suggests that a 50% improvement in the external quantum efficiency of silicon is achievable by using simple multi-layer front-back ITO/metallic and oxide coatings.

## Methods

Optical absorptivity of the multilayer solar cell was measured using the commercial Lumerical Inc. Software^30^ which provides FDTD numerical simulations. All simulations were performed in a 2D environment, where boundary conditions of periodic in horizontal direction and perfectly matched layer (PML) in vertical direction were considered. Spectrums of material properties of ITO, SiO_2_, a-Si, Al_2_O_3_ and Al where taken from the literature^15,31^. A total of 1000 points were uniformly sampled from the input space for training the model, and the corresponding output values (absorptivity) were obtained from FDTD simulations. Running time for these 1000 simulations are equivalent to ~22 direct simulations for absorptivity of distinct geometries when simulated over the wavelength range with 10 nm increments. Neural network modeling was done using an in-house model generation, training and validation code written and implemented in MATLAB.

### Data Availability

The datasets generated during the current study are available from the corresponding author on reasonable request.

## Electronic supplementary material

Supplementary information
