# Supplementary material for: Rapid Optimization of External Quantum Efficiency of Thin Film Solar Cells Using Surrogate Modeling of Absorptivity
Source: Sci Rep. 2018 May 25;8:8170. doi: 10.1038/s41598-018-26469-3 (PMC5970245; doi:10.1038/s41598-018-26469-3)
Supplement: Supplementary file 1 — Supplementary information [file 41598_2018_26469_MOESM1_ESM.docx]

# RAPID OPTIMIZATION OF EXTERNAL QUANTUM EFFICIENCY OF THIN FILM SOLAR CELLS USING SURROGATE MODELING OF ABSORPTIVITY

Mine Kaya^a^, Shima Hajimirza^a^

^a^ Department of Mechanical Engineering, Texas A&M University, 3123 TAMU, College Station, TX, USA 77843-3123

- Mine Kaya

minekaya@tamu.edu

- Shima Hajimirza, **Corresponding Author**

shima.hm@tamu.edu

(979) 845-4280

# SUPPLEMENTARY INFORMATION

# A. Neural Networks

Neural networks convey the effect of inputs to the output by means of a series of functional relationship evaluated at artificial neurons. These neurons could be connected in series (different layers) and in parallel (within the same layer). A layer can have multiple parallel neurons, but the size of the input (output) layer must match the dimension of function input (output). The overall output of the network is mathematically given by:

|  | $\mathbf{p}_{\mathbf{i}}\mathbf{=}f_{i}\left( W_{i}\mathbf{p}_{\mathbf{i-1}} \right), \forall1\leq i\leq L,$  $\mathbf{p}_{\mathbf{0}}\boldsymbol{=}\left[ p_{1} , p_{2},\ldots,1 \right]^{\mathbf{T}}$,  $p_{1}=\hat{z}_{1}\boldsymbol{=}2\frac{z_{1}-z_{1,min}}{z_{1,max}-z_{1,min}}+1.$ | (A.1) |
| --- | --- | --- |

where $L$ is the number of layers, $i$ is layer index, $W$ is the coefficient matrix determined as a result of NN training. $\mathbf{p}_{\mathbf{i}}$ and $f_{i}$ are the output and the transfer function of the $i^{th}$ layer, respectively. In the present study, hyperbolic tangent, $\tanh(\cdot)$ is used in both layers as a transfer function. The details of NN training can be found in references^1,2^. In summary, NN is trained in order to minimize the regularized sum of squared error,

|  | $E\left( \mathbf{v} \right)\boldsymbol{=}\beta\mathbf{e}^{\mathbf{T}}\mathbf{e}\boldsymbol{+}\gamma\mathbf{v}^{\mathbf{T}}\mathbf{v}$, | (A.2) |
| --- | --- | --- |

where $\mathbf{e}=\mathbf{y-}\mathbf{p}_{\mathbf{L}}$ and $\mathbf{v}$ is the vector form of the weight matrix $W$. $\mathbf{v}^{\mathbf{T}}\mathbf{v}$ term is added to the cost function in order to obtain a smoother function response thus avoiding overfitting. $\gamma$ and $\beta$ are the Bayesian regularization parameters set iteratively^1^.

Levenberg-Marquardt algorithm involving the Gauss-Newton approximation for Hessian and gradient of the cost function based on Jacobian is an effective training algorithm providing no second-derivative calculations and always positive-definite Hessian matrix.

|  | $\mathbf{v}_{\mathbf{new}}=\mathbf{v}+\boldsymbol{\Delta v}$,  $\left[ \beta J^{T}J+\delta I \right]\boldsymbol{\Delta v}=-J^{T}\mathbf{e}.$ | (A.3) |
| --- | --- | --- |

$\delta$ is the Marquardt parameter which ensures the positive definiteness of Hessian matrix. The details of Levenberg-Marquardt method implementation on NN training can be found in Hagan et al.^1^.

# B. Simulated Annealing (SA)

Simulated annealing is an optimization algorithm inspired by metal annealing process^3^. At every iteration, a candidate solution $\mathbf{x}_{\mathbf{c}}=\mathbf{x}^{(k)}+\Delta\mathbf{x}^{(k)}$ is chosen randomly from a multivariate Cauchy distribution that becomes narrower as time progresses^4^. Every new candidate is either accepted or rejected based on a probabilistic Metropolis criterion: If the utility function improves (EQE increases), it is accepted, otherwise it is accepted only with the following probability:

|  | $\boldsymbol{p}_{\boldsymbol{k}}\boldsymbol{=1+}\exp\left( \frac{\boldsymbol{\eta}_{\boldsymbol{e}}\left( \mathbf{x}^{\boldsymbol{(k)}} \right)\boldsymbol{-}\boldsymbol{\eta}_{\boldsymbol{e}}\boldsymbol{(}\mathbf{x}_{\boldsymbol{c}}\boldsymbol{))}}{\boldsymbol{c}_{\boldsymbol{1}}\boldsymbol{T}_{\boldsymbol{k}}} \right)\boldsymbol{.}$ | (B.1) |
| --- | --- | --- |

where $T_{k}$ is the so-called temperature of the system, which decays exponentially starting from an initial temperature $T_{k}=T_{o}e^{-c_{1}k}.$ $c_{1}$ and $c_{2}$ were chosen as 1.5 and 2 respectively for the current problem.

# C. Quasi-Newton (QN)

QN is a gradient-based algorithm which searches the best solution iteratively by looking into the direction of the most ascent. The advantage of QN method is that it eliminates the expensive Hessian ($H$) calculation^5^, where $\Delta\mathbf{x}^{(k)}\boldsymbol{=}\mathbf{x}_{\mathbf{c}}-\mathbf{x}^{(k)}$ is found from:

|  | $\Delta\mathbf{x}^{k}\boldsymbol{=}\mathbf{g}^{\mathbf{(k)}}\left[ H^{(k)} \right]^{-1},$ | (C.1) |
| --- | --- | --- |

where $\mathbf{g}^{\mathbf{k}}$ is the gradient of the approximate utility function,

|  | $\mathbf{g}^{\mathbf{k}}=\nabla\hat{\eta}_{e}(\mathbf{x}^{\left( k \right)})$ | (C.2) |
| --- | --- | --- |
|  | $=b{\cdot\eta}_{e}\left( x^{\left( k \right)} \right)\mathbf{e}_{3}\left( 1-\frac{1}{x_{3}} \right)-ae^{-bx_{3}}\int\lambda\frac{\partial\hat{f}\left( \mathbf{x},\lambda\right)}{\partial\mathbf{x}} I\left( \lambda\right)d\lambda.$ |  |

where $\mathbf{e}_{3}=\left( 0,0,1,0,0 \right)^{T}$. Therefore we can compute the gradient vector of the approximate utility function ($\hat{\eta}_{e}$) using the gradient of the surrogate function ( $\hat{f}$). The convenient fact about the above equation is that the partial derivative of $\hat{f}$ can be easily computed using the sensitivity concept in NN by means of back-propagation,

|  | $\frac{\partial\hat{f}}{\partial\mathbf{x}_{\mathbf{n}}}=W_{1}^{T}\mathbf{s}_{\mathbf{i}}.$ | (C.3) |
| --- | --- | --- |

where $\mathbf{s}_{\mathbf{i}}$ is the Marquardt sensitivity^1^,

|  | $\mathbf{s}_{\mathbf{L}}\boldsymbol{=}f_{L}^{'}\left( W_{L} \mathbf{y}_{\mathbf{L-1}} \right)$**,**  $\mathbf{s}_{\mathbf{i}}\boldsymbol{=}f_{i}^{'}\left( W_{i} \mathbf{y}_{\mathbf{i-1}} \right)\left[ W_{m+1} \right]^{T}\mathbf{s}_{\mathbf{i+1}} \forall1\leq i\leq L-1$. | (C.4) |
| --- | --- | --- |

Hessian in equation (C.1) is updated using Broyden-Fletcher-Goldfarb-Shanno (BFGS) formula^5^.

**References**

1. Hagan, M. T., Demuth, H. B., Beale, M. H. & De Jesus, O. *Neural Network Design*. (PWS Publishing Company, 2014). doi:10.1007/1-84628-303-5

2. Foresee, F. D. & Hagan, M. T. Gauss-Newton approximation to Bayesian regularization. in *Proceedings of the 1997 International Joint Conference on Neural Networks* 1930–1935 (1997). doi:10.1109/ICNN.1997.614194

3. Kirkpatrick, S., Gelatt, C. D. & Vecchi, M. P. Optimization by Simulated Annealing. *Science (80-. ).* **220,** 671–680 (1983).

4. Lee, C. Fast simulated annealing with a multivariate Cauchy distribution and the configuration’s initial temperature. *J. Korean Phys. Soc.* **66,** 1457–1466 (2015).

5. Fletcher, R. *Practical Methods of Optimization*. (John Wiley and Sons, 2000).
